# A Rare Case of Retroperitoneal Leiomyosarcoma Presenting as Shortness of Breath

**DOI:** 10.7759/cureus.81100

**Published:** 2025-03-24

**Authors:** Salim Ahmed, Navid Darouian

**Affiliations:** 1 Internal Medicine, University of California, Los Angeles, Los Angeles, USA

**Keywords:** asymptomatic presentation, incidental radiological finding, pelvic retroperitoneal leiomyosarcoma, primary retroperitoneal mass, soft tissue sarcoma

## Abstract

Retroperitoneal leiomyosarcomas (RLMSs) are rare soft tissue sarcomas. These sarcomas often grow silently until compressive symptoms arise, making incidental imaging a common mode of detection. Complete surgical resection remains the cornerstone of treatment, though this can be challenging due to tumor invasion of critical structures.

Here, we report the case of a 78-year-old female with a history of chronic obstructive pulmonary disease (COPD), diabetes, and hypertension who presented with acute shortness of breath. Initial imaging for presumed COPD exacerbation incidentally revealed a large retroperitoneal mass compressing the inferior vena cava (IVC). Subsequent CT-guided core needle biopsy confirmed an RLMS. Given the tumor’s proximity to the IVC and the patient’s severe comorbidities, surgical resection was deemed too high risk. A multidisciplinary tumor board recommended radiation therapy, which she is currently undergoing.

This case exemplifies the typical clinical course of RLMSs, including incidental detection, diagnostic confirmation by core needle biopsy, and challenges in surgical management. The case also highlights the importance of a multidisciplinary approach to manage RLMS and the necessity for specialized care at centers experienced in handling these rare tumors. Advances in targeted therapies, including tyrosine kinase inhibitors, offer future directions in therapy.

## Introduction

Retroperitoneal leiomyosarcomas (RLMSs) are a type of soft tissue sarcoma. Soft tissue sarcomas are a rare and diverse group of cancers that can occur anywhere in the body. Soft tissue sarcomas represent fewer than 1% of adult malignancies, of which approximately 13% originate from the retroperitoneal space. These cancers affect two per one million people [1].

RLMSs are most often diagnosed after the age of 40 years, with the 54-65-year age group being the most affected. Women are more commonly affected than men, with tumors involving the inferior vena cava (IVC) being five times more likely to occur in women compared to men [2].

The large retroperitoneal space typically allows the slow growth of RLMS tumors to a significant size before compressive symptoms occur. Masses, therefore, are typically clinically silent. Symptoms that do occur later, are due to tumor compressive symptoms and are non-specific. These include abdominal pain, fullness, heaviness, shortness of breath, reflux, constipation, and leg swelling [1].

Diagnosis usually starts when RLMS is found incidentally on imaging. CT or MRI abdominal imaging typically shows large retroperitoneal tumors compressing, invading, or spreading metastatically to nearby structures and organs. Imaging features can be variable. Masses identified on imaging, therefore, have a large differential diagnosis. Given the morbidity and mortality associated with RLMS, correct identification is critical, and core needle biopsy is necessary to confirm tissue diagnosis [3]. The cornerstone of management is complete resection of the RLMS tumor with intact capsule and negative margins. Surgical resection is often challenging due to the mass invading the nearby critical structures. Other treatment options include anthracycline-based chemotherapies with or without adjuvant radiation therapy, though survival and quality of life show little benefit without surgery [4]. Postoperative radiation following complete resection has the best outcomes, with a five-year survival of 57% versus a 40% survival without surgery. Preoperative radiation has also been proposed and is discussed later in this article.

Here, we present the case of a 78-year-old woman found to have RLMS. Her presentation resembles many of the typical features described above.

## Case presentation

A 78-year-old female with a history of chronic obstructive pulmonary disease (COPD) (on 2 L oxygen by a nasal cannula at baseline), asthma, diabetes, hypertension, and hyperlipidemia presented to a community hospital with two days of acutely worsening shortness of breath. She had presented to the emergency room (ER) the day prior, treated for an acute COPD exacerbation, and sent home with a Medrol dose pack and azithromycin. Because of continued dyspnea, she presented back to the ER the next day. During this second evaluation, the ER team completed a CT angiogram of her thorax, which did not show a pulmonary embolism, but did show a right upper lobe pneumonia. Her physical examination was notable for diffuse wheezing and rales in the right upper lung fields. Labs were notable for a leukocytosis of 26 x 10^9^/L with a left shift. She did not have any chest pain, troponin, B-type natriuretic peptide lab abnormalities, or electrocardiography findings to suggest a cardiac cause for her symptoms. She was started on empiric intravenous (IV) antibiotics, IV steroids, and nebulizer treatments with albuterol and ipratropium around the clock for presumed community-acquired pneumonia and COPD exacerbation. Her respiratory symptoms began to improve that day and continued to improve the days thereafter.

**Figure 1:**
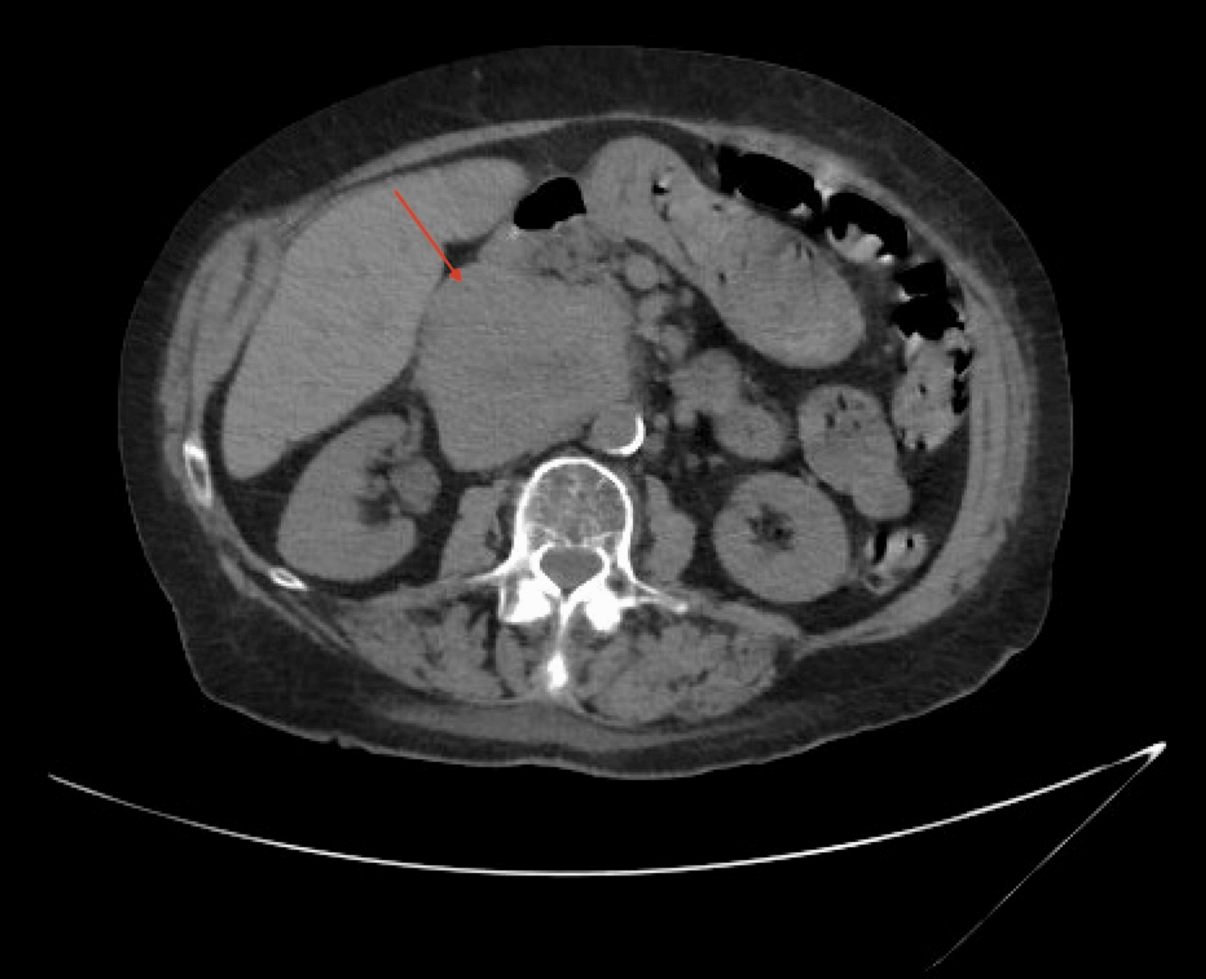
CT of the abdomen/pelvis with contrast showing a large retroperitoneal mass compressing the inferior vena cava.

During her initial workup, there was an incidental finding that would later become the focus of her hospitalization. The CT thorax demonstrated a new, large, unknown abdominal mass. This was followed up with dedicated abdominal CT, which confirmed a large right retroperitoneal mass of unclear etiology, compressing, and possibly invading the IVC. It is worth mentioning that IVC compression can cause lower extremity edema and abdominal distention, but the patient did not have these findings. IVC compression typically does not cause respiratory symptoms, unless a pulmonary embolism ensues, but this was ruled out with a CT angiogram of the thorax. Furthermore, the mass was not large enough to cause any restrictive effect on the lungs to contribute to her dyspnea. A CT image of the mass compressing the IVC is shown in Figure 1.

**Figure 2:**
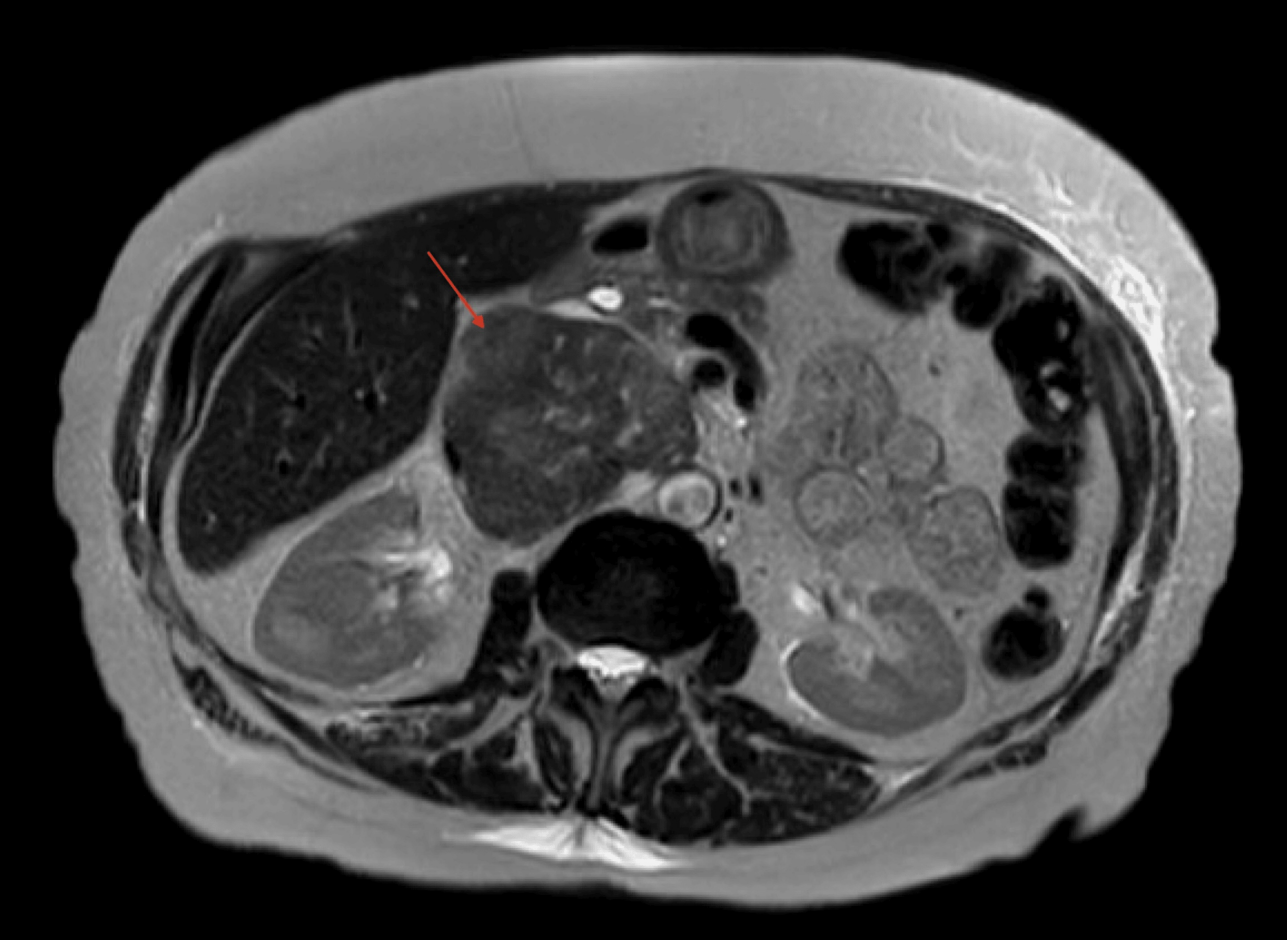
Follow-up MRI of the abdomen confirming a large right-sided retroperitoneal mass, possibly invading the inferior vena cava.

The retroperitoneal mass was confirmed on follow-up imaging with an MRI of the abdomen, as shown in Figure 2.

The imaging findings raised concern for malignancy, possibly a lymphoma versus sarcoma. Oncology was consulted and recommended a CT-guided core needle biopsy, which later confirmed a grade 1 RLMS. Surgical oncology was consulted in-house, but given the surgical risk due to possible IVC invasion and her oxygen-dependent COPD, surgery was not recommended. The patient then followed up as an outpatient with the UCLA sarcoma service, with surgical oncology, radiation oncology, and the tumor board consulted. The multidisciplinary team agreed that the surgery risk outweighed its benefit. Radiation therapy was recommended. The patient is currently undergoing radiation therapy.

## Discussion

We present a case of a rare neoplasm, RLMS. This case possesses many classic presentations of this rare cancer. While the patient presented with shortness of breath, which can be seen with RLMS, the patient’s underlying COPD and concomitant pneumonia may have confounded that symptom. Her malignancy was found incidentally on imaging, as typically occurs, and confirmed with a core needle biopsy. Her cancer was deemed not resectable, unfortunately, and she is currently undergoing radiation therapy.

This case reinforces several important elements in the management of this rare condition. Once a mass is identified, whether on palpation or incidental imaging, it is important to have follow-up cross-sectional imaging to determine the extent of the lesion and its proximity to critical structures. This was certainly true with this case, given its proximity to the IVC. Furthermore, fine-needle aspiration is insufficient to establish a diagnosis. Core needle biopsy is required [5]. These features were present in this case.

Once RLMS is accurately diagnosed, management can be complicated. Although surgery is the mainstay of treatment, it must be planned appropriately. The preoperative evaluation considers the extent of the disease, the patient’s functional performance, and the healthcare team responsible for the patient’s care [6]. First, the extent of the disease is assessed by staging via chest, abdomen, and pelvic CT. This is crucial to determine the tumor’s extent. If metastasis has occurred, as often occurs to the liver and lungs, chemotherapy becomes first-line therapy [6]. Second, attention should be paid to the patient’s comorbidities. This is important to assess the patient’s fitness for an extensive surgery. In addition, an assessment of the patient’s nutritional status is needed to improve postsurgical outcomes, reduce complications, and enhance quality of life post-procedure. Third, patients with RLMS should be managed at centers with surgical oncologists, medical oncologists, radiation oncologists, pathologists, and radiologists with expertise in sarcomas. One estimate suggests that a minimum of 10 to 13 RLMS cases annually are needed to maintain competency in managing these complex and rare tumors [6].

It should be noted for completeness that surgical resection is possible even when RLMS invades the IVC, as occurred in this case. The approach to surgery is driven by the level of tumor involvement at the IVC and the presence of collateral venous networks. Surgical options include partial resection with cavoplasty and complete resection with graft placement and ligation [7]. In our case, while the patient was managed at a center experienced in RLMS, she was not an ideal surgical candidate due to existing comorbidities, and surgery was not recommended.

For local tumors that are not resectable, radiation therapy is recommended, as occurred in this case. However, the evidence supporting radiation therapy for non-resectable RLMS remains uncertain [8]. The STRASS1 trial evaluated preoperative radiation therapy before surgery compared to surgery alone for a subgroup of patients with retroperitoneal sarcoma (RPS). It found that high-quality radiation therapy may improve control of RPS in select histologies of disease [8]. Current research is investigating different radiation therapy modalities for unresectable RPS cases. For metastatic RLMS, first-line therapy consists of anthracycline or gemcitabine-based chemotherapy regimens; however, these regimens portend a modest survival duration of 14-16 months [9].

Recent advances in molecular profiling have advanced knowledge of the pathophysiology driving leiomyosarcoma leading to several novel therapeutics. Newer treatments target DNA damage repair pathways and metabolism related to oncogenesis [10]. One such treatment is the tyrosine kinase inhibitor, anlotinib. It has been studied to show non-inferiority to conventional first-line chemotherapies [10]. While new treatments are encouraging, first-line chemotherapy agents have had limited success, underscoring the difficulty in treating RLMS.

## Conclusions

RLMS is a rare malignancy, typically affecting adults above the age of 40 years. Cases involving the IVC, as seen in this case, occur five times more often in women compared to men. The disease is often clinically silent and found incidentally on imaging. A core needle biopsy is needed to confirm the diagnosis. The gold standard of treatment is complete surgical resection with negative margins. However, this is not always possible due to patient comorbidities and tumor involvement in adjacent structures. Non-operative cases are managed with radiation therapy or chemotherapy, though evidence supporting either modality is limited. Research is underway to explore new treatment options, such as the tyrosine kinase inhibitor, anlotinib. Regardless of the treatment option chosen, it is important to manage cases with a multidisciplinary team of medical and surgical oncologists, radiologists, and pathologists at a center experienced in managing this rare disease.
